# Gene Electro Transfer of Plasmid Encoding Vascular Endothelial Growth Factor for Enhanced Expression and Perfusion in the Ischemic Swine Heart

**DOI:** 10.1371/journal.pone.0115235

**Published:** 2014-12-29

**Authors:** Barbara Hargrave, Robert Strange, Sagar Navare, Michael Stratton, Nina Burcus, Len Murray, Cathryn Lundberg, Anna Bulysheva, Fanying Li, Richard Heller

**Affiliations:** 1 Frank Reidy Research Center for Bioelectrics, Old Dominion University, Norfolk, Virginia, United States of America; 2 School of Medical Diagnostics and Translational Sciences, College of Health Sciences, Old Dominion University, Norfolk, Virginia, United States of America; 3 Naval Medical Center Portsmouth, Portsmouth, Virginia, United States of America; 4 Sobran, Inc. Fairfax, Virginia, United States of America; Thomas Jefferson University, United States of America

## Abstract

Myocardial ischemia can damage heart muscle and reduce the heart's pumping efficiency. This study used an ischemic swine heart model to investigate the potential for gene electro transfer of a plasmid encoding vascular endothelial growth factor for improving perfusion and, thus, for reducing cardiomyopathy following acute coronary syndrome. Plasmid expression was significantly greater in gene electro transfer treated tissue compared to injection of plasmid encoding vascular endothelial growth factor alone. Higher gene expression was also seen in ischemic versus non-ischemic groups with parameters 20 Volts (p<0.03), 40 Volts (p<0.05), and 90 Volts (p<0.05), but not with 60 Volts (p<0.09) while maintaining a pulse width of 20 milliseconds. The group with gene electro transfer of plasmid encoding vascular endothelial growth factor had increased perfusion in the area at risk compared to control groups. Troponin and creatine kinase increased across all groups, suggesting equivalent ischemia in all groups prior to treatment. Echocardiography was used to assess ejection fraction, cardiac output, stroke volume, left ventricular end diastolic volume, and left ventricular end systolic volume. No statistically significant differences in these parameters were detected during a 2-week time period. However, directional trends of these variables were interesting and offer valuable information about the feasibility of gene electro transfer of vascular endothelial growth factor in the ischemic heart. The results demonstrate that gene electro transfer can be applied safely and can increase perfusion in an ischemic area. Additional study is needed to evaluate potential efficacy.

## Introduction

Myocardial ischemia occurs when myocardial oxygen delivery fails to satisfy the rate of myocardial oxygen consumption. Given the high metabolic demand of the myocardium, ischemia can have immediate effects on the ability of the heart to contract effectively. When ischemia persists, permanent heart damage may result [1]. Current treatment for myocardial ischemia is directed at improving the balance between oxygen delivery and consumption. In some pathological conditions that lead to myocardial ischemia, only medication is necessary to decrease the rate of myocardial oxygen consumption relative to the rate of myocardial oxygen delivery. However, in cases of acute coronary syndrome, the imbalance can be so abrupt and so severe that more aggressive therapy is necessary. Traditionally, patients with suitable anatomies following atherosclerotic changes can be treated with percutaneous catheter based interventions (PCI) or with surgical coronary artery by-pass (CABG). These treatments aim to restore adequate blood flow through the coronary arteries and, therefore, to deliver adequate myocardial oxygen.

Unfortunately, the anatomy of some patients' atherosclerotic disease limits the potential for PCI and/or CABG to improve myocardial oxygen delivery to ischemic areas of the heart [2]. Although mechanical ventricular assist devices and cardiac transplant are possibilities at some time points, options are limited once patients have progressed to end-stage heart failure. Currently, only transmyocardial laser revascularization (TMLR) offers potential improvement in myocardial oxygen delivery in the native heart in these patients. TMLR is thought to work indirectly by up-regulating the expression of vascular endothelial growth factor (VEGF) and promoting collateral neovascular growth, which improves perfusion.

Gene therapy is an approach that has emerged as a potential method for delivering angiogenic factors such as VEGF to the ischemic myocardium directly. Adenoviral (Ad) delivery has provided useful and thought provoking results [3]. However, Ad-based vectors are limited because this approach may cause severe tissue damage with large areas of interstitial inflammatory cell infiltration and myocyte necrosis [3]. Currently, clinical trials are investigating the efficacy of injecting naked DNA directly into the heart. Although the concept is promising, it is unclear whether the level of expression achieved will be clinically effective. Fortuin et al. reported clinical improvements in patients in a 1-year follow-up study after direct myocardial gene transfer of VEGF-2 using naked plasmid DNA; however, no angiographic evidence of angiogenesis was reported [4].

Gene electro transfer (GET) is an approach that utilizes electric fields to facilitate intracellular delivery of molecules [5], [6]. The controlled application of electric pulses facilitates the entry of DNA by permeabilizing the cell membrane. This approach has been successfully used *in vivo* to deliver plasmid DNA to skeletal muscle [6] and to cardiac tissue [7], [8]. Previously, we and others demonstrated that electro transfer could be used effectively to deliver plasmid DNA directly to cardiac tissue *in vivo* [7]–[10]. We have further confirmed that electro transfer is a viable approach for delivering plasmid DNA into cells of the porcine heart *in vivo*. The GET parameters were selected based on the size and structure of swine cardiac myocytes. We also determined a pulse length that could be administered during the rising phase of the R wave of the electrocardiogram (ECG), but concluded before the start of the T wave, while at the same time providing significant gene expression. We previously demonstrated that synchronizing the electric pulses to the rising phase of the R wave of the ECG allowed for efficient administration of GET with minimal risk of fibrillating the heart. [9].

Previously, we reported that various electrode configurations can be used to facilitate gene delivery to the heart [10]. In this study, we used our custom-made 7 mm electrode and injected DNA at a concentration of 0.2 mg/100 µl. Using this electrode, we evaluated in vivo delivery of a plasmid encoding vascular endothelial growth factor (pVEGF) to the myocardium of the heart via varying field strengths.

VEGF is a potent mediator of angiogenesis [11]–[13]. Humans express alternately spliced isoforms of VEGF-A varying from 121, 145, 165, 183, 189, to 206 amino acids in length [12]. VEGF_165_ appears to be the most abundant and potent isoform, followed by VEGF_121_ and VEGF_189_ [11]–[13]. VEGF has also been associated with angiogenesis following myocardial infarction [14]. Gene therapy approaches that have been utilized for treating cardiac ischemia have shown that VEGF plays a role in angiogenesis [4], [15].

In the present study, we investigated the ability of GET to deliver pVEGF effectively to the ischemic swine heart. We further evaluated the influence that this approach had on the level of perfusion within the ischemic area of the heart. The results obtained in this study demonstrate that GET is an effective tool for delivering plasmid DNA to the heart. Delivery of pVEGF utilizing the appropriate GET parameters resulted in increased perfusion within the ischemia area of the heart.

## Materials and Methods

### Animals

Seventy, adult, castrated male, domestic farm pigs weighing 31–47 kg were used for this study. All swine were purchased as specific pathogen free (SPF) for pseudorabies and brucellosis from a commercial swine vendor's (Bellview Farms, Smithville, Virginia) closed herds. All animals were given health examinations by an ACLAM board certified veterinarian and were determined to be free of disease. In addition, all animals were processed on-site and were quarantined for a 7 day acclimation period before any procedures were conducted. An ultrasound exam was conducted on each animal's heart to obtain baseline measures of cardiac performance. Animals were housed in stainless steel runs, with three animals to a room and one animal per run to allow socializing. The animal rooms were maintained in accordance with the *Guide for the Care and Use of Laboratory Animals* [16], including a 12:12-hour light cycle. Room temperature was maintained at 15–17°C with a relative humidity between 40–65%, and with 12–15 air changes hourly. Animals were feed Swine food (Teklad Swine Chow, no. 8753, Madison, WI, USA) twice per day and provided water ad libitum via an automatic watering system (Edstrom Industries, Waterford, WI, USA). Environment enrichment included daily food treats, toys, and human interaction. This study was conducted in an AAALAC-accredited facility and in compliance with the Animal Welfare Act and other federal statutes and regulations pertaining to animals [16]. All procedures outlined in this study were approved by Old Dominion University's animal care and use committee.

### Plasmid

The plasmid utilized for these experiments was pVax1-hVEGF_165_ (pVEGF). The plasmid was prepared commercially (Aldevron, Fargo, ND) and suspended to a concentration of 2 mg/ml in sterile injectable saline. Each production of plasmid was tested by Aldevron using a *Limulus* Amebocyte Lysate assay to assure endotoxin levels were <0.1 EU/µg plasmid.

### Pre-surgical preparation

All animals were sedated by intramuscular administration of ketamine (20 mg/kg) and diazepam (3–5 mg/kg). Sedation facilitated placement of catheters in veins of both ears for all intravenous injections. Once the catheters were in place, the animals were anesthetized with isoflurane through a nose mask. Once anesthetized, each animal was intubated with a 5 or 6 French endotracheal tube and placed on a ventilator.

### Surgical preparation

Surgical preparation was reported previously [9]. Briefly, animals were placed in a surgical plane of anesthesia by administering isoflurane at 2–3% with an oxygen flow rate of 2 liters per minute. The lungs were ventilated (200–300 ml tidal volume with 14–18 breaths per minute) using a BonAir mechanical ventilator (DRE, Inc., Louisville, KY, USA). Animals were monitored constantly and the level of isoflurane was adjusted as needed to maintain the animal in a surgical plane of anesthesia. Additional preparation included shaving the ventral thorax (chest) and using an initial surgical scrub. The animals were placed in a dorsal recumbence position on a heated surgical table. ECG leads were attached and the ECG R wave was monitored for the purpose of delivering the electric field. The chest was once again scrubbed with 70% alcohol and chlorhex-Q.

### Surgical procedure

A sterile surgical field was established by draping the chest. The skin and underlying soft tissues were divided sharply at the midline. A striker saw was used to open the sternum, and a standard sternal retractor was used to expose the pericardium, which was opened from the diaphragmatic reflection to the level of the atrium. Amiodarone (300 mg) was given intravenously (i.v.) and a lidocaine drip (50–75 mg/kg/min, i.v.) was used to provide protection from arrhythmia. ECG and blood pressure were monitored continuously using a SurgiVet monitor (Smiths Medical Dublin, OH). A blood pressure cuff was placed around the right hind limb to continuously assess blood pressure. Body temperature was continuously measured by placing a temperature probe in the rectum, with the probe also being connected to the SurgiVet monitor. Blood gases were measured using a Vetstat Electrolyte and Blood Gas Analyzer (IDEXX, Westbrook, Maine).

### Creation of Ischemia

Partial occlusion of blood flow through the left anterior descending coronary artery (LAD) was achieved by placing a 4-0 silk suture around the LAD approximately 3–4 cm from the apex of the left ventricle. A 20 gauge blunt needle was placed over the vessel and the suture was tied securely around the shaft of the blunt needle. Once the suture knot was in place, the blunt needle was removed, creating a partially occluded coronary stenosis. Four silk sutures were placed 2 cm apart on the anterior wall of the left ventricle both to mark the location of each plasmid injection and to define the treatment area of ischemic myocardium, which was supplied by the stenotic LAD. Assessment of coronary perfusion of this treatment region was performed using the SPY Intraoperative Perfusion Assessment System. (See “SPY Analysis” below).

### Treatment procedure

**Figure 1:**
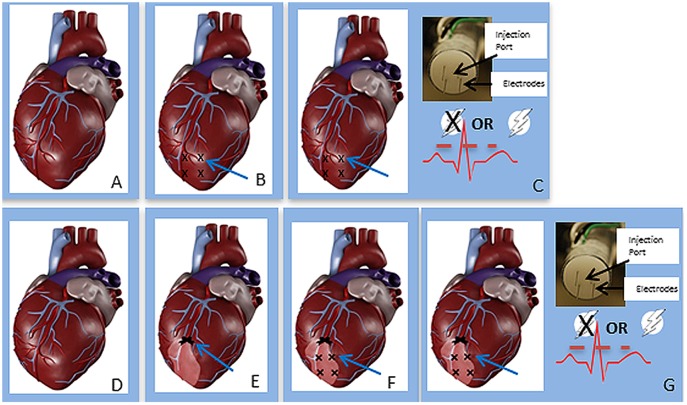
Sequence for administration of therapy. A) The heart was exposed as described in the Methods section under “surgical procedure”. B) The left anterior descending coronary artery is ligated with a permanent suture (arrow) and a SPY image is collected to visualize change in perfusion. C) Sutures are placed to designate the treatment sites. D) Sequence of treatment, applicator containing 4 electrodes and injection port is placed at the treatment site marked by suture; 100 µl of plasmid solution at a concentration of 2 mg/ml is injected; 8 pulses are administered. Procedure is repeated for the other 3 sites.

The treatment protocol was initiated after occlusion of the LAD. Fig. 1 shows a schematic of the treatment procedure. Animals were divided into three basic treatment groups: no treatment, injection of pVEGF without GET, or injection of pVEGF with GET. We had established previously that the pulses could be administered without causing fibrillation by synchronizing the electric pulses to the rising edge of the R wave, with the pulse being completed before the start of the T wave [9]. Therefore, prior to initiating the pulsing protocol, the ECG for each animal was analyzed to delineate the rising phase of the R wave. The electrode applicator contained a central injection port, which allowed the injection needle to be inserted to a controlled depth. To target delivery to the myocardium, a spacer that allowed insertion of the injection needle to 3.5 mm was used. The applicator contained 4 penetrating electrodes spaced 5 mm apart and 7 mm long to target delivery to the myocardium of the left ventricle [8]. Several GET parameters were tested. For those animals receiving GET with pVEGF, the following procedure was implemented. The pulse width was constant at 20 milliseconds, while the applied electric field was varied. The delivery parameters included 20 V, 40 V, 60 V or 90 V. A total of four sites, each 2 cm apart, on the anterior wall of the left ventricle of the porcine heart were injected with plasmid and exposed *in vivo* to the applied electric fields (Fig. 1). The applicator forms a 5×5 mm area. This electrode was designed to facilitate the administration of four pulses in each of two perpendicular directions [8]. For animals receiving only an injection of pVEGF without GET (injection only, or IO), the procedure was the same in terms of the placement of applicator and DNA injection, except that no electric pulses were administered. For the no treatment group (sham), we followed the same procedure except that the applicator was not used, no pVEGF was injected, and GET was not performed.

For all animals, after the last site was treated, the sternum was closed with surgical steel monofilament 18-inch surgical needles. All other incisions were closed with Vicryl plus-antibacterial suture (3.5 metric). The surgical area was bandaged using sterile TelFa non- adherent pads and secured by wrapping the chest with Vet Wrap to protect the incision from contamination. Postoperatively, all animals remained in the operating room under constant observation until extubation. Analgesics for pain relief included Carprofen, given intramuscular (IM) just prior to completion of the surgical procedure (4 mg/kg) and once daily (100 mg tablets by mouth) for 3 days. Buprenorphine (0.1 mg/kg) was also given IM once after the animal was extubated and breathing comfortably on its own.

### SPY Analysis

The SPY Intraoperative Perfusion Assessment System (Novadaq Technologies, Inc, Bonita Springs, FL) was used to image, capture, and view dynamic fluorescence images of the area of myocardium at risk. Fluorescent intensity was measured in arbitrary units. Changes in the intensity of fluorescence have been shown to be directly related to changes in perfusion [17]. Assessment of fluorescent intensity was performed on the treatment area before and after ischemia was induced by partial LAD stenosis and again 2 weeks post infarction. To produce an image, an intravenous injection of isocyanogreen (ICG) was given at 10 µl/kg. ICG is a sterile, water soluble, tricarbocyanine dye. Before injection, ICG was reconstituted using the aqueous solvent provided with the ICG.

### Cardiac Enzyme Assays

Cardiac troponin-1 and creatine kinase were measured using commercially available kits. The cardiac enzyme troponin-1 was measured using a Pig Cardiac Troponin-I, K-ASSAY Pig Cardiac Troponin-I, High Sensitive ELISA (Kamiya Biomedical Company, Seattle, WA) according to manufacturer's instructions. Creatine kinase was measured using a Pig CK-MM ELISA (Kamiya Biomedical Company, Seattle, WA) also according to manufacturer's instructions. Three milliliters of whole blood were collected immediately after chest closure and 1 hour, 3 hours, 1 day, 2 days, 4 days and 14 days post procedure; blood samples were centrifuged and plasma removed and frozen at −80°C until running troponin and creatine kinase ELISAs. Coated wells of the kits were loaded with 100 µl of calibrator or diluted samples. All samples were run in duplicate. The optical density was read at 450 nm with a microtiter plate reader within 5 minutes of adding the stop solution.

### Echocardiography

All animals were sedated with diazepam (50 mg, PO) and ketamine (20 mg/kg, IM), to alleviate anxiety before being placed under general anesthesia (isoflurane in oxygen). A left side, short axis, left ventricular parasternal electrocardiographic approach was used to analyze selected aspects of heart function. A 180 Plus Sonosite ultrasound machine (National Ultrasound, Duluth, Georgia) with a 7 Hz transducer was used to perform two dimensional and M-Mode echocardiography. Analysis software, which included a cardiac package, was used to quantitate ejection fraction (EF), cardiac output (CO), left ventricular end diastolic volume (LVEDV), left ventricular end systolic volume (LVESV) and stroke volume (SV). The software in this machine uses Teicholz measures obtained from the M mode to calculate the variables analyzed. Electrocardiography was performed at three different time points as follows: before the LAD was partially occluded, following closure of the chest cavity (post myocardial infarction (MI)), and 14 days post infarction.

### Tissue Harvesting

Two, 7 or 14 days post MI, animals were placed under a surgical plane of anesthesia and the sternum re-opened. For animals 2 or 7 days post MI, the heart was exposed; the animal was euthanized; and the heart was removed. For animals 14 days post MI, the heart was exposed; the SPY procedure was performed as described above; the animal was then euthanized and the heart removed. For all animals, once the heart was removed, areas of the left ventricle defined by the sutures were dissected and frozen at −80°C until analysis for the presence of vascular endothelial growth factor (VEGF).

### VEGF Expression

VEGF expression was measured using the Quantikine Human VEGF Immunoassay ELISA kit (R&D Systems, Inc. Minneapolis, MN 55413). This is a solid phase ELISA designed to measure VEGF_165_ levels in the supernatant of the heart. It contains Sf 21-expressed recombinant human VEGF_165_ and antibodies raised against the recombinant protein. The kit includes plates pre-coated with a monoclonal antibody specific for VEGF. Tissue samples were homogenized and standards and samples were pipetted into the wells. Any VEGF present was bound by the immobilized antibody. After washing away any unbound substances, an enzyme-linked polyclonal antibody specific for VEGF was added to the wells. Following a wash to remove any unbound antibody-enzyme reagent, a substrate solution was added to the wells, and color developed in proportion to the amount of VEGF bound in the initial step. The color development was stopped and the intensity of the color was measured.

## Results

It should be noted that the echocardiographic results reported in this study were obtained in male swine that were in a deep plane of anesthesia.

### GET delivery of pVEGF to the beating heart

**Figure 2:**
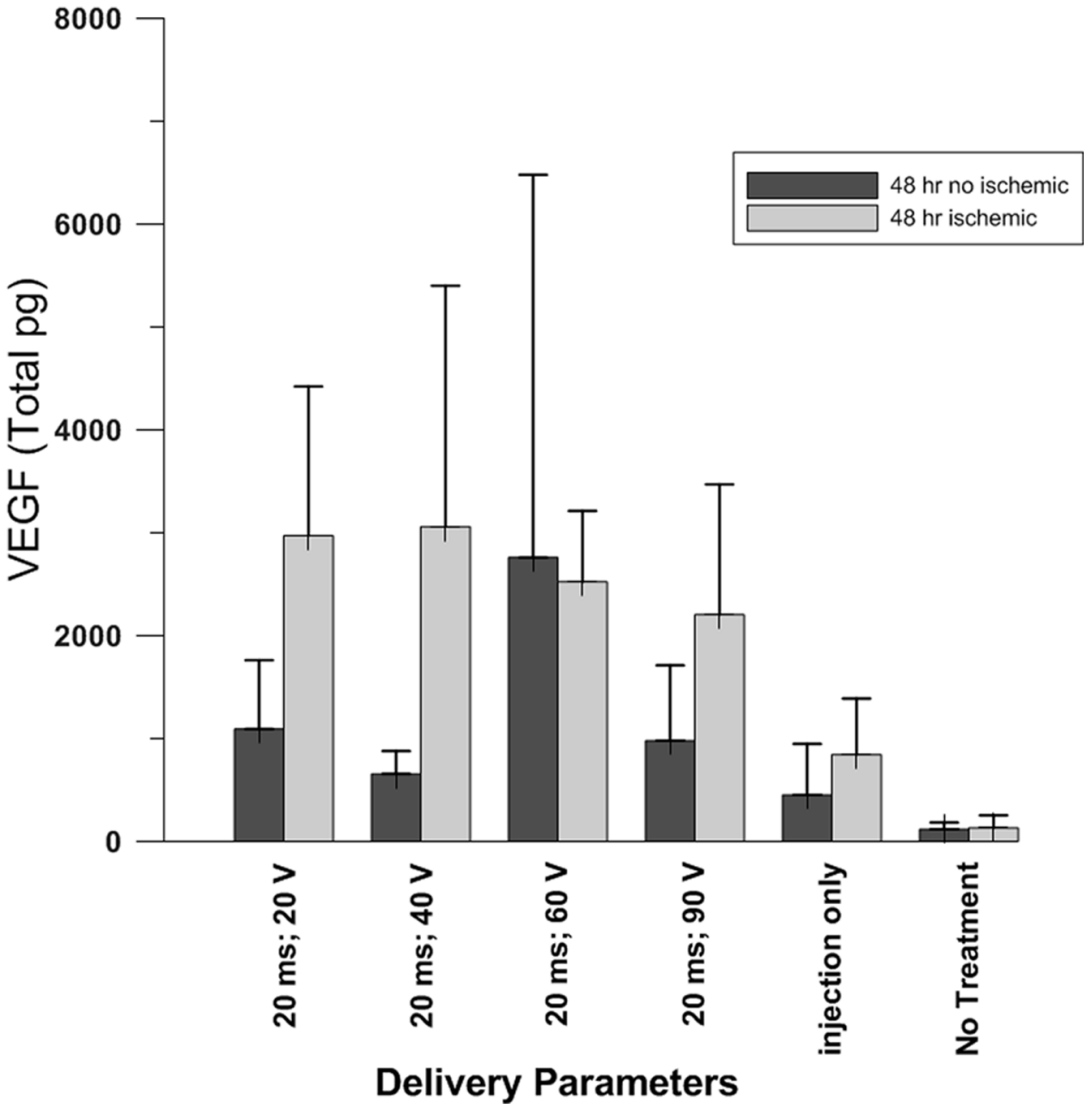
Intra-cardiac delivery of pVax1-hVEGF_165_. The duration of intra-cardiac delivery of pVax1-hVEGF_165_ was 20 milliseconds at varying voltages. Plasmid expression was significantly greater in the ischemic hearts with parameters 20 milliseconds; 20 V (p<0.03 non- ischemic versus ischemic), 20 milliseconds; 40 V (p<0.05 non- ischemic versus ischemic), and 20 milliseconds; 90 V (p<0.05 non- ischemic versus ischemic), but not with the 20 milliseconds; 60 V parameter (p<0.09 non- ischemic versus ischemic). The non-treated hearts had significantly less expression (p<0.002) than the hearts treated with injection only.

This procedure was performed following either partial occlusion of the LAD (ischemic tissue) or without occlusion of the LAD (non-ischemic tissue). Each animal had four treatment sites on the anterior surface of the left ventricle. All GET parameters were kept constant (20 millisecond pulse width, 8 pulses and 5 millimeter electrode gap), except for the applied voltage. Four different voltages (20, 40, 60 and 90) were used to deliver pVEGF. After treatment, the animal was returned to its housing facility for 48 hours. Expression levels of VEGF were significantly greater if the plasmid was delivered by GET versus by DNA IO or sham treatment (p<0.05). In addition, expression was significantly greater (p<0.05) in ischemic hearts than in non-ischemic hearts when GET was administered at 20 V (p<0.03), 40 V (p<0.05) and 90 V (p<0.05), but not with the 60 V parameter (p<0.09). The non-ischemic hearts receiving only pVEGF and no GET had significantly less gene expression (p<0.002) than did the hearts treated with injection of plasmid and electroporation (Fig. 2).

**Figure 3:**
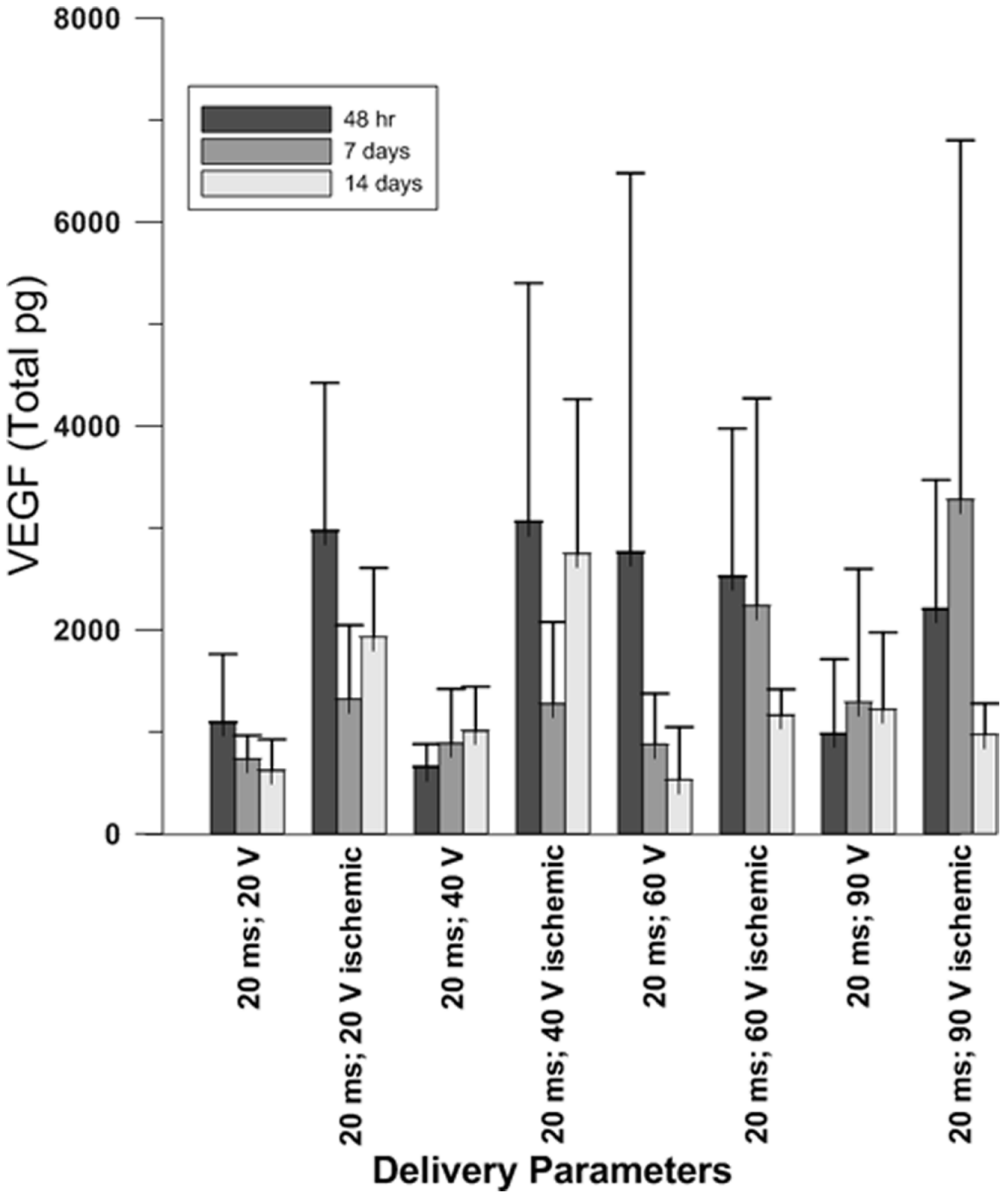
Expression of pVax1-hVEGF_165_. pVEGF expression was analyzed over a two week period at 48 hours, 7 days and 14 days. Peak expression was observed at 48 hours in all ischemic tissues at all parameters except 20 milliseconds; 60 V. VEGF expression was significantly greater in the 20 milliseconds; 60 V parameter 14 days after ischemia was produced.

We also investigated changes in pVEGF expression over time (Fig. 3) in the ischemic and non-ischemic hearts. As was seen at 48 hours, expression was significantly greater when the plasmid was delivered by GET versus DNA IO or with sham treatment (p<0.05). When comparing the difference in expression between delivery to ischemic versus non-ischemic tissue, pVEGF expression was significantly greater in the ischemic hearts at the 48 hour time point in all parameters except 60 V (20 V p<0.03, 40 V p<0.05 and 90 V p<0.03). At 14 days post-infarction, VEGF expression was significantly greater in the 20 V (p<0.001), 40 V (p<0.03) and 60 V (p<0.0001), but not in the 90 V.

### Analysis of Perfusion by SPY

The SPY Intraoperative Perfusion Assessment System was used to image, capture and view fluorescence images for the visual assessment of fluorescent intensity of the treatment area on the anterior myocardium. After partial occlusion of the mid LAD, the drop in fluorescent intensity occurring in the ischemic portion of the left ventricle was determined prior to initiation of treatment and immediately after the LAD was partially occluded. A drop in fluorescent intensity is an indirect marker that correlates directly with perfusion. The mean drop in intensity of fluorescence from the pre-MI period to immediately after the MI was between 82–87% (Table 1). Two weeks post MI and post treatment, the mean drop in fluorescent intensity was between 19–71%. In the hearts treated with pVEGF + GET, levels of improvement varied based on the GET parameters used (Table 1). The largest improvement was observed when pVEGF was delivered with GET administered at 60 V.

**Table 1:** Fluorescent Intensity measurement using The SPY Elite Intraoperative Perfusion Assessment System.

GROUP	% Decrease In Fluorescent Intensity Immediately Post- MI Compared to Pre-MI	% Decrease In Fluorescent Intensity 2 Weeks Post-MI Compared to Pre-MI
No Treatment (n = 4)	86.8±2.9	71±22.2
pVEGF Injection Only (n = 4)	84.3±10.5	64.3±24.9
pVEGF +GETP (20 ms; 20 V) (n = 5)	82.8±6.7	61.6±16.3
pVEGF + GET (20 ms; 40 V) (n = 5)	83.6±14.1	33.2±16.9
pVEGF + GET (20 ms; 60 V) (n = 5)	87.2±4.2	19.0±42.9**
pVEGF + GET (20 ms; 90 V) (n = 5)	83.2±6.7	51.2±3.5

pVEGF  =  plasmid vascular endothelial growth factor.

**p<0.03 GET-pVEGF (20 milliseconds; 60 V) versus No Treatment or pVEGF Injection Only. ms  =  milliseconds, V  =  volts. Data stated as mean ± SD.

This result suggests that delivery with GET administered at 60 V was the most appropriate. This observation also indicates that it may be important to have elevated levels of expression in both ischemic and non-ischemic tissue. We draw this conclusion because GET delivery of pVEGF administered at 60 V performed in either ischemic or non-ischemic tissue achieved similar expression levels (except at Day 14). Therefore, this parameter was used to investigate effects of pVEGF delivery on changes in echocardiography and in cardiac enzymes further.

### Echocardiography

Echocardiography was performed with all animals under general anesthesia (isoflurane and oxygen). It was used to assess ejection fraction (EF), cardiac output (CO), stroke volume (SV), heart rate (HR) and left ventricular end diastolic and systolic volumes (LVEDV and LVESV). Echocardiography was performed on all animals before surgery, after partial occlusion of the LAD coronary artery and closure of the chest cavity, and 14 days post MI at the left ventricular mid-cavity level using the left side short axis parasternal view. The myocardial infarction established a new baseline value. Changes in these parameters were assessed by comparing the measurements taken at 2 weeks to the baseline levels pre and post-MI.

**Figure 4:**
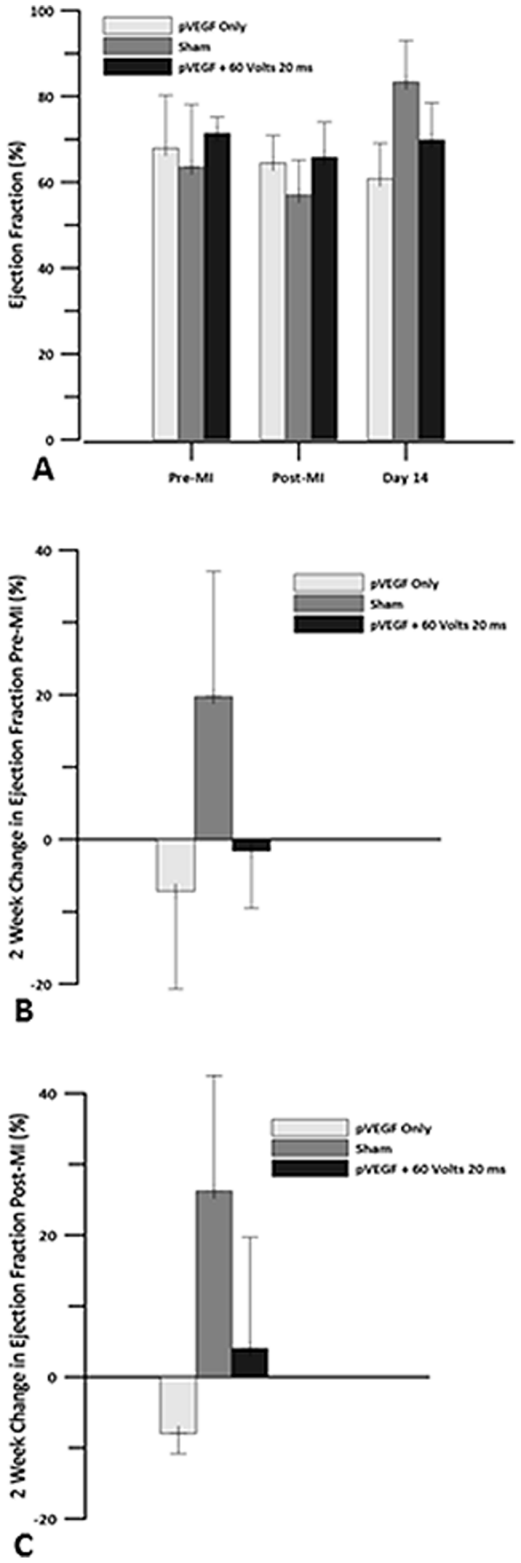
Ejection Fraction. Echocardiography was used to obtain levels of ejection fraction for pVEGF injection only, sham and pVEGF +GET groups. A) Ejection fraction levels before MI, after the left anterior descending artery (LAD) was occluded and 14 days post MI. B) Change in ejection fraction at 2 weeks when compared to pre MI baseline. C) Change in ejection fraction at 2 weeks when compared to post MI baseline. There were no statistically significant differences in EF between the groups.

In the GET treated group, EF was relatively stable. There was a decrease immediately post-MI, but after two weeks, EF had returned close to baseline (pre-MI) level (Fig. 4). The sham group had a decreased EF immediately post-MI, but had a large increase compared to pre-MI baseline at 2 weeks. IO group had a decreased EF immediately post-MI and did not recover (i.e., the EF level remained reduced). There was a significant difference between the sham and the IO treated animals (p<0.03).

**Figure 5:**
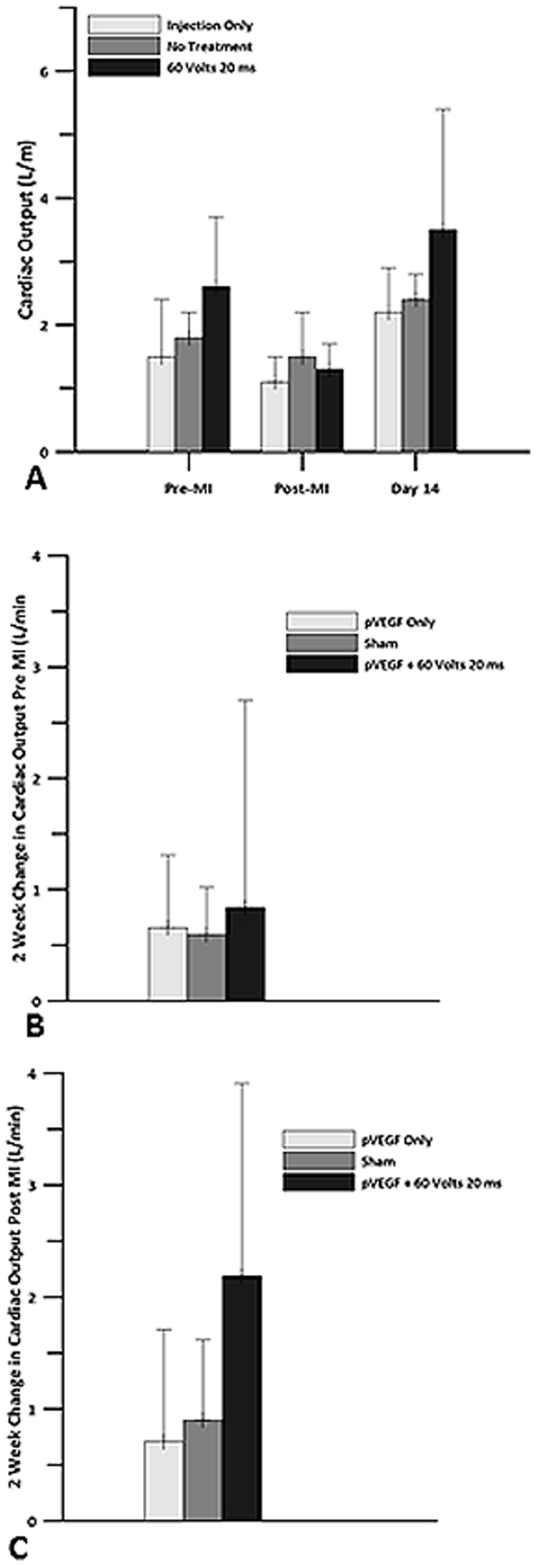
Cardiac Output. Echocardiography was used to obtain cardiac output measurements for pVEGF injection only, sham and pVEGF +GET groups. A) Cardiac output levels before myocardial infarction, after the left anterior descending artery (LAD) was occluded and 14 days post MI. B) Change in cardiac output at 2 weeks when compared to pre MI baseline. C) Change in cardiac output at 2 weeks when compared to post MI baseline. There were no statistically significant differences in CO between the groups.

Changes in CO for all three groups were similar. Immediately post-MI, all three groups had a reduction in CO. Comparing 2 weeks vs immediately post-MI, there was a 0.71 L/min increase in CO in the IO group, a 0.90 L/min increase in CO in the sham group, and a 2.2 L/min increase in the plasmid + GET treated group two weeks post infarct (Fig. 5). There were no significant differences between the groups.

**Figure 6:**
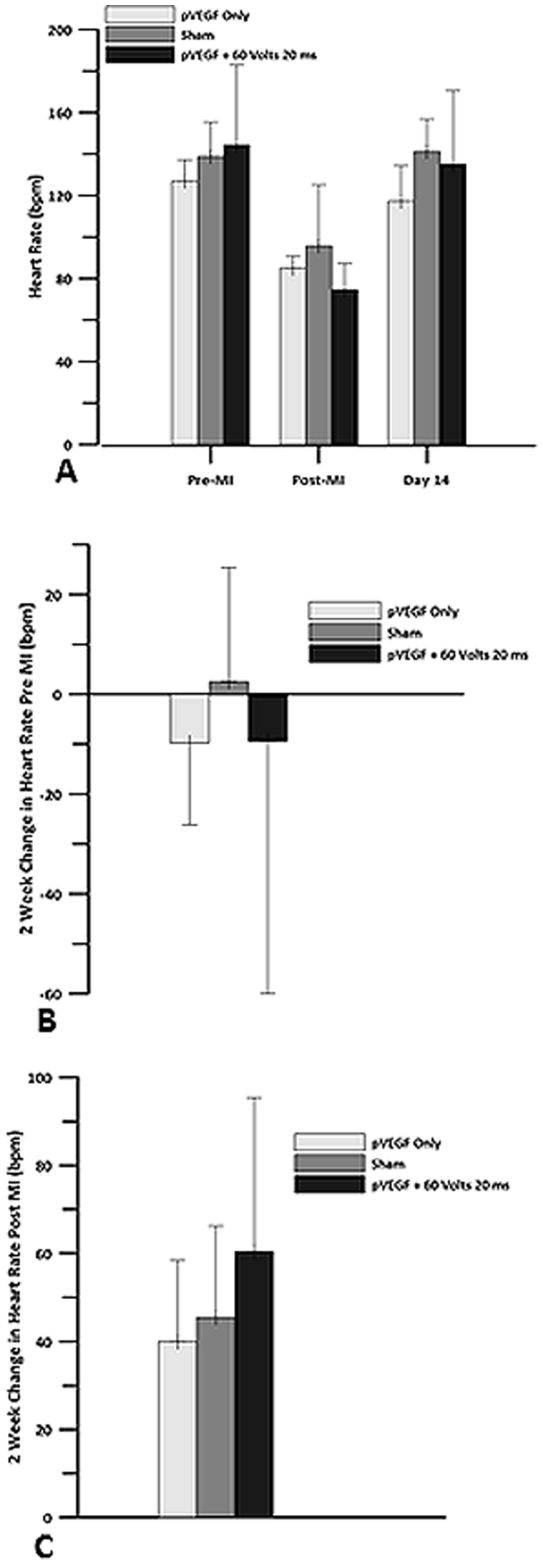
Heart Rate. Echocardiography was used to obtain heart rate for pVEGF injection only, sham and pVEGF +GET groups. A) Heart rate before myocardial infarction, after the left anterior descending artery (LAD) was occluded and 14 days post MI. B) Change in heart rate at 2 weeks when compared to pre MI baseline. C) Change in heart rate at 2 weeks when compared to post MI baseline. There were no statistically significant differences in HR between the groups.

Measurements of HR at 2 weeks compared to pre-MI revealed minor changes, with IO and GET groups showing a small decrease and sham showing a small increase. Comparisons at 2 weeks with immediately post-MI revealed that HR increased by 40 bpm in the IO group, by 45.5 bpm in the sham group, and by 64.4 bpm in the plasmid +GET group. There were no significant differences between the groups (Fig. 6).

**Figure 7:**
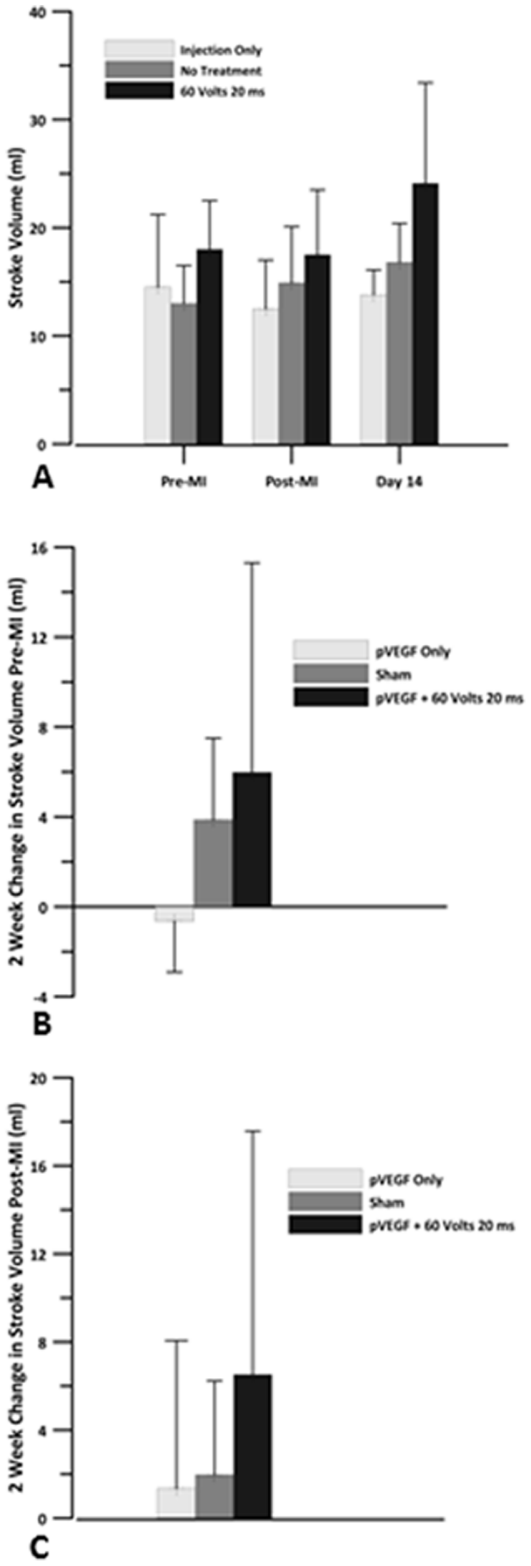
Stroke volume. Echocardiography was used to obtain the stroke volume for pVEGF injection only, sham and pVEGF +GET groups. A) Stroke volume before myocardial infarction, after the left anterior descending artery (LAD) was occluded and 14 days post MI. B) Change in stroke volume at 2 weeks when compared to pre MI baseline. C) Change in stroke volume at 2 weeks when compared to post MI baseline. There were no statistically significant differences in SV between the groups.

Two weeks post-MI, SV was slightly decreased in the IO group and increased in the sham and GET groups when compared to pre-MI (Fig. 7). The increase was higher in the GET group compared to the sham group. Stroke volume at 2 weeks was higher than SV immediately post-MI for all three groups. GET group had the highest increase while IO and sham had only a small increase. There were no significant differences between the groups.

**Figure 8:**
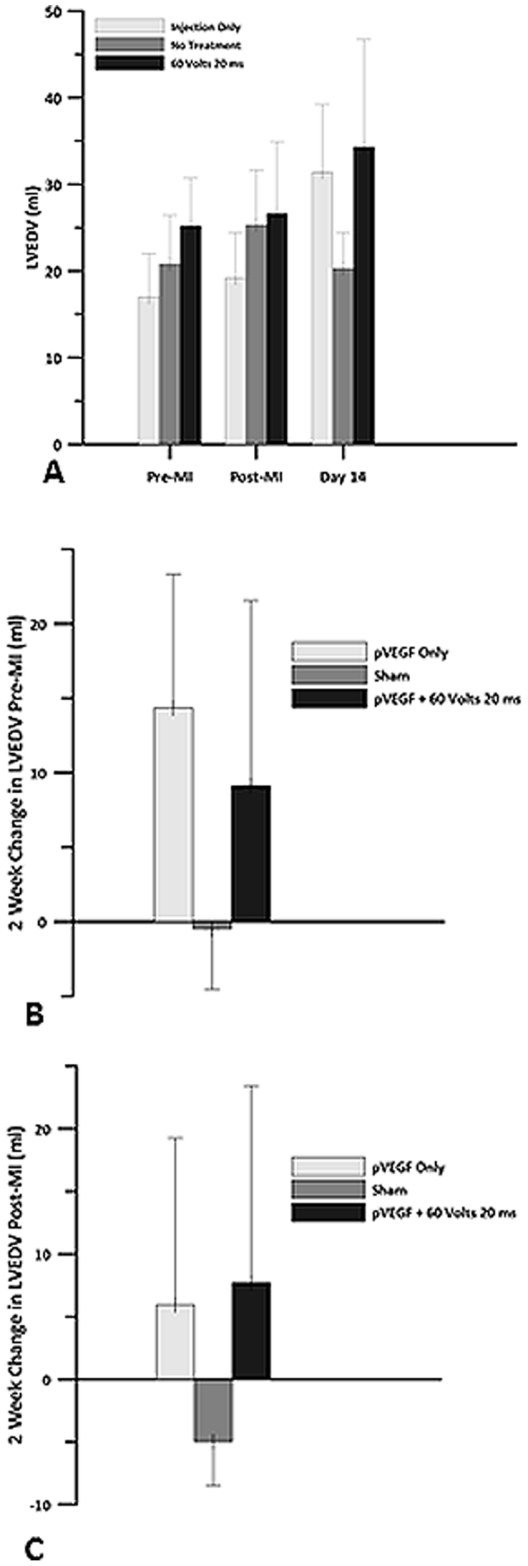
Left ventricular end diastolic volume. Echocardiography was used to obtain left ventricular end diastolic volume (LVEDV) for pVEGF injection only, sham and pVEGF +GET groups. A) LVEDV before myocardial infarction, after the left anterior descending artery (LAD) was occluded and 14 days post MI. B) Change in LVEDV at 2 weeks when compared to pre MI baseline. C) Change in LVEDV at 2 weeks when compared to post MI baseline. There were no statistically significant differences in LVEDV between the groups.

**Figure 9:**
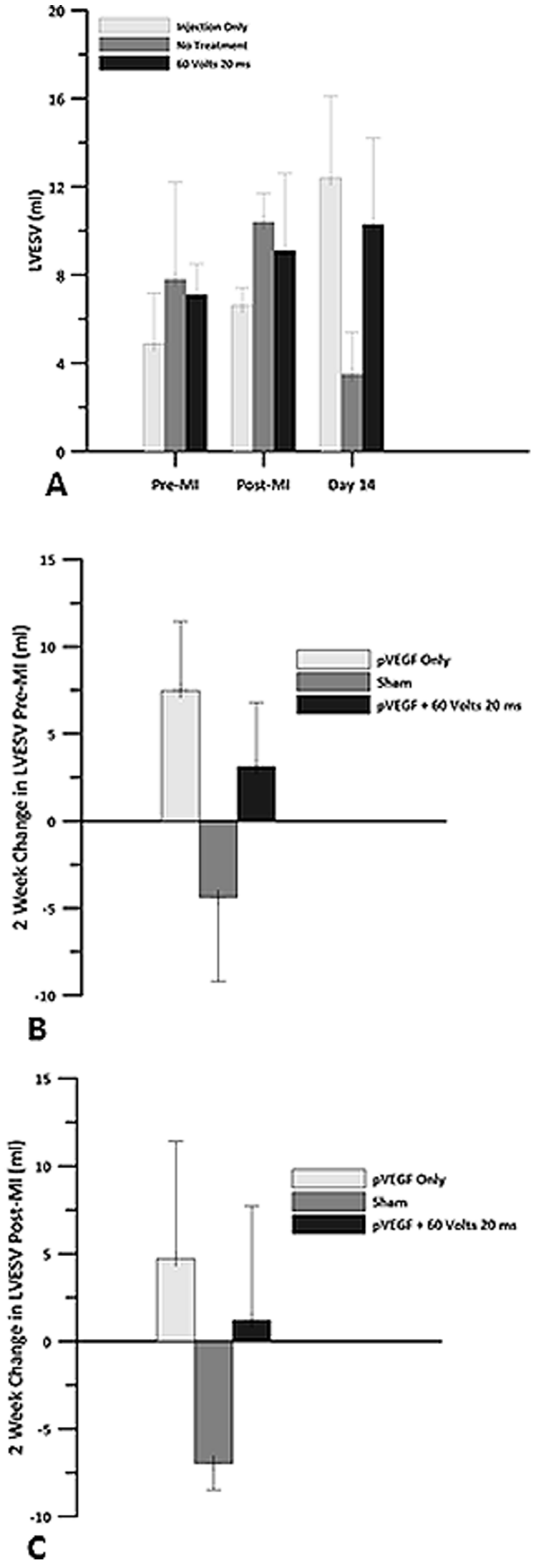
Left ventricular end systolic volume. Echocardiography was used to obtain left ventricular end systolic volume (LVESV) for pVEGF injection only, sham and pVEGF +GET groups. A) LVESV before myocardial infarction, after the left anterior descending artery (LAD) was occluded and 14 days post MI. B) Change in LVESV at 2 weeks when compared to pre MI baseline. C) Change in LVESV at 2 weeks when compared to post MI baseline. There were no statistically significant differences in LVESV between the groups.

There was an increase in LVEDV from 19 ml post MI to 31 ml 2 weeks post MI in the IO group, a decrease from 25 ml to 20 ml in the sham group, and an increase from 27 ml to 34 ml in the plasmid +GET group. These changes did not differ significantly (Fig. 8). There was an increase in LVESV from 7 ml immediately post-MI to 12 ml 2 weeks post MI in the IO group, a decrease from 10 ml to 3 ml in the sham group, and an increase from 9 ml to 10 ml in the plasmid +GET group (Fig. 9). There were no significant differences between the groups.

### Analysis of Cardiac Enzymes

Plasma cardiac troponin levels were elevated 1 day after partial occlusion of the LAD in all animals (Table 2), suggesting adequate ischemic stimulus in all hearts. However, in the pVEGF +GET group, troponin levels had returned to levels below those observed immediately after the chest was closed. This was not observed in the pVEGF only group or in the sham group. The increase in creatine kinase (Table 2) was observed immediately after the chest was closed in all animals. However, enzyme levels in the pVEGF +GET group were 80% lower by Day 14 post infarction compared to a 60% drop in the pVEGF only group at the same time point. Venous catheter malfunctions prevented us from obtaining values at Day 14 post MI in the sham animals. These data suggests that exposure of the cardiac myocytes to GET yields minimal and transient cardiac damage.

**Table 2:** Plasma troponin and creatine kinase measurements before and after occlusion of the LAD coronary artery.

Plasma Troponin				Creatine Kinase			
Time	pVEGF + GET (20 ms; 60 V)	pVEGF injection only	Sham	Time	pVEGF + GET (20 ms; 60 V)	pVEGF injection only	Sham
Pre MI	3.52±3.3 (ng/ml)	1.8±1.7 (ng/ml)	4.0±5.2 (ng/ml)	Pre MI	409±289 (ng/ml)	456±408 (ng/ml)	696±206 (ng/ml)
Post MI	59±42	26±23	31±16	Post MI	2210±1604	2521±305	1363±348
1 hour	31±35	9.6±11	21±8	1 hour	3682±1968	4740±2338	2187±273
3 hours	157±124	140±151	133±90	3 hours	4488±2038	7289±3926	5246±1093
1 day	6772±2396*	3339±1295	3980±1600	1 day	4361±122	9202±4610	6776±5143
2 days	5789±2873#	2144±347	2259±1232	2 days	5072±4759	3235±1975	1498±1022
4 days	1024±1117	369±53	554±37	4 days	538±238		429±63
14 days	10.7±0	11.9±0		14 days	420±106	1004±408	

Enzyme values immediately after LAD occlusion was used as the baseline value. Data stated as mean ± SD.

*p<0.02 pVEGF+GET (20 milliseconds; 60 Volts) 1 day post operatively versus pVEGF injection only or Sham.

# p<0.009 pVEGF+GET (20 milliseconds; 60 Volts) 2 days post operatively versus pVEGF injection only or Sham.

## Discussion

Insufficient myocardial oxygen delivery will result in ischemic cardiomyopathy. This condition can, in turn, be associated with adverse cardiac remodeling and reduced long-term survival [18]. In cases where PCI and CABG are not applicable secondary to poor coronary artery anatomy, a novel approach, utilizing gene transfer of angiogenic factors, may improve myocardial perfusion. In this study, naked pVEGF was injected into the myocardium of the beating swine heart. The injection was followed by GET in some hearts to investigate the effects of enhancing expression of pVEGF in the ischemic and non-ischemic swine heart. The intra-cardiac injection of pVEGF +GET increased the expression of pVEGF compared to an injection of plasmid alone. In addition, with some GET parameters, expression was elevated significantly in the ischemic swine heart compared to levels in the non-ischemic tissue. Expression was increased significantly 48 hours after treatment in all but one parameter used and remained elevated for a longer period of time than was seen with pVEGF injection alone.

SPY Intraoperative Perfusion Assessment System, was used to evaluate fluorescent intensity as an indicator of perfusion within a specific region of the left ventricle. Delivery of pVEGF with 20 V or 90 V did not improve fluorescent intensity compared to IO or sham. Animals that were treated with pVEGF +GET at 40 V were observed to have improved fluorescence intensity from an 83% drop post MI (pretreatment) to a 33% drop 2 weeks after treatment (Table 1). We observed that animals treated with pVEGF+ GET at 20 milliseconds at 60 V exhibited the largest improvement in fluorescent intensity 2 weeks after treatment. The decrease in fluorescent intensity improved from an 87% drop to only a 19% drop. This change in fluorescent intensity after two weeks correlates with a significant (p<0.03) increase in coronary blood flow in animals treated with pVEGF +GET at 60 V, when compared to the IO or sham treated animals. This finding suggests that angiogenesis may be occurring. To confirm that the increased perfusion was related to angiogenesis, it would have been useful to have sectioned the ischemic tissue and performed immunostaining for angiogenic markers (i.e. CD31). Unfortunately, the samples were homogenized to perform the ELISAs to determine the expression levels of VEGF. Therefore, the ability to do histological analysis on the samples was lost. Now that there is evidence that the level of perfusion in the ischemic swine heart can be influenced, future plans are to do a more comprehensive study that would include histological analysis.

We used parasternal short axis left ventricular M mode echocardiographic recordings to assess aspects of left ventricular function and structure [19]. M mode echocardiographic measurements of left ventricular function are reported to be less accurate than hemo-dynamically derived estimations [20], [21]. However, others have found a good correlation between angiographic and echocardiographic left ventricular dimensions and functions [22], [23].

Analysis of the changes in LVEDV and LVESV, using the newly established post MI baseline values shortly after occlusion of the LAD indicated a 31% increase in LVEDV, which was associated with a 71% increase in LVESV in the IO group. There was a 20% decrease in LVEDV and a 67% decrease in LVESV in the sham group. In the plasmid +GET group of animals, we observed a 29% increase in LVEDV, which was associated with a 13% increase in LVESV. We speculate that these data may suggest a problem with diastolic filling in the IO and sham treated animals. In the IO animals, an increase in preload to the left ventricle (LVEDV) was not associated with the expected decrease in LVESV. In fact, LVESV actually trended upward. In the sham group, preload decreased as did LVESV, suggesting a possible alteration in filling of the left ventricle.

We speculate that a loss of compliance, or stiffening of the left ventricle after ischemia, was greater in the hearts treated with plasmid only or saline as evidenced by the relationships exhibited between LVEDV and LVESV. Conditions such as myocardial infarction can cause the heart muscle to enlarge abnormally and/or become stiff [24]. A stiffened left ventricle would make the filling process during diastole less efficient and could have led to the decrease in LVEDV observed in the sham animals 2 weeks post MI. It should be remembered that this group of animals had a 46% increase in EF 2 weeks post MI. Therefore, the high EF in the sham group may be due to improper or reduced filling of the chamber, which forced the ventricle to pump a higher fraction of the total amount of blood entering it. This decreased left ventricular blood volume remaining in the chamber would necessitate an increase in EF to maintain cardiac output.

The increased EF in this group may reflect a greater percent of the reduced volume being pumped out of the left ventricle. In the plasmid +GET group, the increase in preload was accompanied by a smaller increase in LVESV, suggesting that contractility of the left ventricle was more efficient than in the other two groups. It is possible for damage to the left ventricle, which is a major pumping chamber, to occur without an abnormal ejection fraction [25]. Hence, the plasmid +GET treated animals may have had a more compliant left ventricle. There was a 2.2 L/min increase in CO in the plasmid +GET group compared to the 0.71 L/min increase in the IO group and to the 0.9 L/min increase in the sham group two weeks post infarction. The plasmid +GET hearts were able to increase HR by 60 bpm and SV by 6.5 ml to sustain this CO. However, HR only increased by 40 bpm and SV by 1.35 ml in the IO group, and by 45.5 bpm and 2.0 ml, respectively, in the sham group. These data suggest reasons for the relatively low CO in the IO and sham groups, although EF was either within normal range (IO group) or high range in the sham group. We have not attempted to make any functional statements regarding these data. However, collectively they may suggest that left ventricular compliance is less in the sham and IO groups than in the group treated with pVEGF +GET.

Changes in cardiac output were supported by the changes in intensity of fluorescence, corresponding to an increase in perfusion. As measured by the SPY System pVEGF +GET (20 ms; 60 V), treated hearts had a greater improvement in fluorescence intensity 14 days post MI than did the IO or sham groups. The improvement in fluorescent intensity in the pVEGF +GET (20 ms; 60 V) treated hearts was 55% greater than in the no treatment group, and 62% greater than in the pVEGF injection only group, suggesting that perfusion was enhanced.

In summary, we have demonstrated that GET enhances the expression of pVEGF and the expression can be maintained for at least 14 days. In addition, delivery and subsequent expression of pVEGF is associated with a greater improvement in fluorescent intensity (coronary perfusion) of the ischemic myocardium in the swine heart.
